# Use of the chronic illness research recruitment taxonomy to evaluate recruitment strategies in an eHealth feasibility study.

**DOI:** 10.1016/j.conctc.2024.101420

**Published:** 2024-12-27

**Authors:** Rosalynn C. Austin, Anne Marie Lunde Husebø, Hege Wathne, Marianne Storm, Kristin H. Urstad, Ingvild Morken, Bjørg Karlsen

**Affiliations:** a Department of Public Health, Faculty of Health Sciences, University of Stavanger, 4036 Stavanger, Norway; b Department of Cardiology, Portsmouth Hospitals University NHS Trust, Portsmouth, PO6 3LY UK; c National Institute of Health and Care Research (NIHR) Applied Research Collaboration (ARC) Wessex, Southampton, SO17 1BJ UK; d Research Group of Nursing and Health Sciences, Research Department, Stavanger University Hospital, Stavanger, Norway; e Department of Quality and Health Technologies, Faculty of Health Sciences, University of Stavanger, 4036 Stavanger, Norway; f Faculty of Health Sciences and Social Care, Molde University College, Molde, Norway; g Avdeling for Helsetjenesteforskning (HØKH), Akershus Universitetssykehus HF, Molde, Norway

**Keywords:** Max 6, Research recruitment strategies, Feasibility trials, Mixed methods, Chronic illness research, eHealth, Remote patient monitoring

## Abstract

**Background:**

Chronic illness research has many challenges making research recruitment difficult. Despite reports of facilitators and barriers to research recruitment challenges remain. The reporting of research strategies and their impact on recruitment and subsequent randomised control trials is not sufficient. A newly developed chronic illness research recruitment taxonomy (CIRRT) details factors and elements observed to impact recruitment around the components of Project, People, and Place. This paper aims to use the chronic illness research recruitment taxonomy to report and evaluate the recruitment strategies, impact they had on recruitment, and alterations to an eHealth feasibility study.

**Methods:**

Retrospective mixed method approach was used to inductively code the research team meeting minutes during the recruitment period. The coding was then abductively matched to the chronic illness research recruitment taxonomy and gaps in the CIRRT noted. Dated coding data were integrated with recruitment progress to explore the impact of research recruitment strategies.

**Results:**

Meeting minutes (n = 66) were analysed, recruitment strategies identified and matched to CIRRT. The reporting and identification of the recruitment strategies was aided by CIRRT use. By integrating the codes that aligned with CIRRT with recruitment progress was observed to be impacted by staffing and researcher visits.

**Conclusions:**

CIRRT may be a useful tool in the evaluation and reporting of research recruitment strategies. Altering the roles of nurses involved and researcher visits to recruiting sites may positively impact on chronic illness research recruitment.

## 1 Introduction

Recruitment to chronic illness research is difficult and many studies do not reach intended recruitment goals. A review of publicly funded studies in the UK found that 56 % (across multiple chronic illness) of clinical trials reached their intended recruitment goal [1]. Recruitment is time consuming, costly, and difficult [[1], [2], [3], [4], [5]]. Recruitment that is neither inclusive nor sufficient has implications for research generalizability [[5], [6], [7]]. Research in chronic illness has additional barriers due to healthcare service access [8] and lower research inclusion rates of older adults, women, and minority ethnic groups [9,10].

Research studies or trials often encounter recruitment challenges resulting in simultaneous adaptations to research recruitment strategies (e.g., altering inclusion criteria, creating more marketing material, increasing collaborations, etc) [6,11]. Concurrent adjustments complicate the assessment of those changes [6,11]. Further, reporting of alterations to subsequent randomised control trials recruitment strategies remains suboptimal [4,11]. The QuinteT [6,12,13] offers a tool for real-time evaluation and intervention around improving research recruitment. This tool needs to be embedded into a trial from early stages requiring substantial funding and research work. A newly developed chronic illness research recruitment taxonomy (CIRRT) with practical questions may therefore offer a possible solution. This CIRRT was developed as the results of a previous restricted review on factors related to research recruitment (conducted by authors of this article) [14]. In this article the CIRRT was used to retrospectively explore the recruitment strategies in a feasibility study This is the first time CIRRT has been used to report on recruitment strategies.

**Fig. 1:**
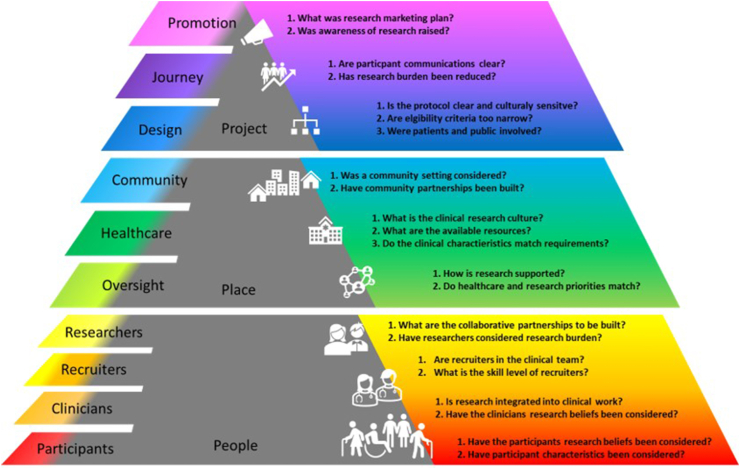
CIRRT taxonomy overview with practical questions from [14].

The CIRRT described in the previous literature review, offers a detailed taxonomy for research recruitment, along with practical questions to guide the reporting and evaluation of research recruitment strategies (Fig. 1) [14]. It identifies and characterises research recruitment components of *people, place*, and *project*. Within each component further factors and elements are described to impact research recruitment. The *people* component encapsulates recruitment strategies which are built with consideration to individuals involved in the research process. The *place* component includes recruitment strategies which are built around the environment where the research occurs, and the *project* component incorporates recruitment strategies which are built around the study design [14]. While the importance and role of a feasibility study to assess both the proposed intervention and research processes (e.g., recruitment) is well established [15], determining the impact of recruitment strategies or alterations remains challenging.

An eHealth intervention entitled eHealth @ Hospital -2- Home (eHealth@H2H) [16] was developed using the MRC complex interventions framework [17] to design and test the feasibility of a nurse assisted remote patient monitoring mobile health (mHealth) intervention for people with chronic illness. The eHealth@H2H intervention was built on literature reviews focusing on patients with heart failure (HF) [18] and colorectal cancer (CRC) [19] together with input from patients and healthcare providers [20]. The feasibility study [21] aimed to test the acceptability and usability of the intervention [22]. The research teams’ original goal was to recruit a total of 30 patients with HF and CRC in a 6-month period.

This research is a sub-study of the eHealth@H2H feasibility study which reports retrospectively on recruitment strategies. The recruitment to the feasibility study for the eHealth@H2H project encountered two main challenges: 1) multiple waves of the COVID19 pandemic and 2) recruiter staffing and role definitions. While recruitment to the feasibility study was successful, it took double the predicted time. The existing ethics approval from the Norwegian Centre for Research Data (NSD) (ID.NO: 523,386) and the participating hospitals’ Privacy Appeals Board stands for this sub-study and followed the declaration of Helsinki [23].

The aim of this paper was use CIRRT to retrospectively report and evaluate the recruitment strategies and their impact on recruitment, in the eHealth@H2H feasibility study.

## 2 Methods

Retrospective analysis of recruitment to the eHealth@H2H feasibility study using CIRRT practical questions and taxonomy (Fig. 1). Research project team meeting minutes were used as qualitative data for this study together with the dates for participant recruitment as quantitative data. The data were independently analysed and integrated by a researcher (RCA). A complementary mixed method (proposed by Palinkas, Mendon and Hamilton [24]) where integration the different data types highlights findings not observed independently [24]. The author of the meeting minutes (BK) reviewed the analysis of data and research team members (RCA, BK, HW, KU) discussed and refined of the findings.

### 2.1 Data source

Meeting minutes between Feb 2021–Dec 2022 were examined retrospectively line by line for reported activities that could be considered as related to research recruitment. These meetings were conducted weekly with the objective of planning and executing the feasibility study. Meeting minutes followed a template design and were written by the same researcher (BK). Minutes were distributed to meeting participants for proof-reading and approval. Final meeting minutes were translated from Norwegian to English by RCA using MS Word translation functionality [25]. The recruitment activity (dates and numbers of people who consented) was used to describe the recruitment progress.

### 2.2 Data analysis

For the qualitative analysis, first CIRRT practical questions (Fig. 1) were used to guide reflexive discussions with the members of the research team (BK, HW, and KH) to summarise the recruitment strategies used. Their answers were put aside until after the inductive coding was finished by RCA. Those answers were used to help with the deductive fitting of the inductive codes to the CIRRT.

RCA coded translated minutes, using line by line using inductive coding [26] to identify any activities related to recruitment in the feasibility study. RCA was not present during recruitment to the eHealth@H2H feasibility study. After coding was completed BK, who was present for the recruitment and author of the meeting minutes, reviewed the coding to sense check and identify any gaps or misrepresentations due to translation in the coding. Agreement was reached on the coding, the codes were abductively tested for fit to CIRRT [14]. Each code was then organised into the taxonomy and re-labelled to match the taxonomy wording. Any codes that did not fit the taxonomy were noted and left unchanged. Coding with exemplar quotes are provided in the supplementary data (LINK to supplementary file 1).

For the quantitative analysis, the dates for participant recruitment were used to create a study recruitment timeline. That timeline was then integrated with finalised coding (anchored by dates from meeting minutes and aligned with CIRRT) to explore for any observable impact in recruitment numbers. Codes which matched peaks or plateaus in recruitment numbers were overlayed onto the recruitment timeline. Finally, the meeting minutes were examined the research team for how these alterations of the recruitment strategy were applied to the subsequent randomised control trial.

## 3 Results

During the eHealth@H2H feasibility study, 66 meeting minutes were identified and analysed. The recruitment strategies observed in the meeting minutes will be presented using CIRRT core components of Project, People, and Place with their embedded factors and elements to characterise observed recruitment strategies.

### 3.1 Recruitment strategies identified in the CIRRT component *project*

The *project* component of CIRRT (Fig. 1) was considered and evaluated by answering 7 practical questions (see Table 1). Exemplar coding with data quotes is provided in supplemental material 1 (LINK to supplementary file 1).

**Table 1:** Practical questions and answers for project component of CIRRT.

Practical questions	Answers
Design	
1) Is the protocol clear and culturally sensitive?	Study design used MRC framework; documents, and questionnaires only in Norwegian.
2) Are the eligibility criteria too narrow?	Thought to be broad, but further broadened during the study.
3) Were patients and public involved?	A user advisory board (UAB) was created with regular meetings.
Journey	
1) Are participant communications clear?	Patient facing documents edited by UAB and an animated video in English and Norwegian created to explain the research participation.
2) Has research burden been considered?	Considerations were made for participants (e.g., equipment and training provided) and clinical collaborators (e.g., token gift cards and training).
Promotion	
1) What was the research marketing plan?	Multi-faceted program for promoting the research (e.g., animated film and poster).
2) Was awareness of the project raised?	Regular collaboration meetings planned, and which was adapted during the study.

**Fig. 2:**
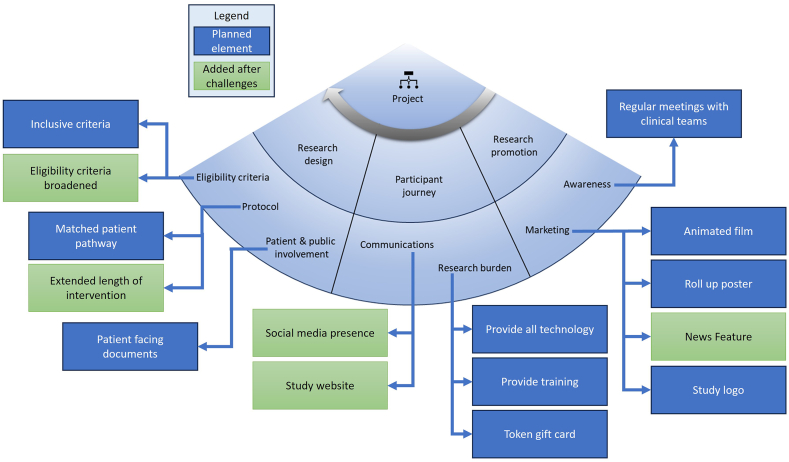
Strategies used in the recruitment component *project* of CIRRT [14]. The planned strategies are in blue boxes and the added strategies are in green boxes.

Findings from the analysis of the meeting minutes provided more details to the practical questions. Coding results are highlighted in Fig. 2 to demonstrate the recruitment strategies (blue boxes) used as well as the alterations (green boxes) made due to challenges in the conduct of the study.

**Fig. 3:**
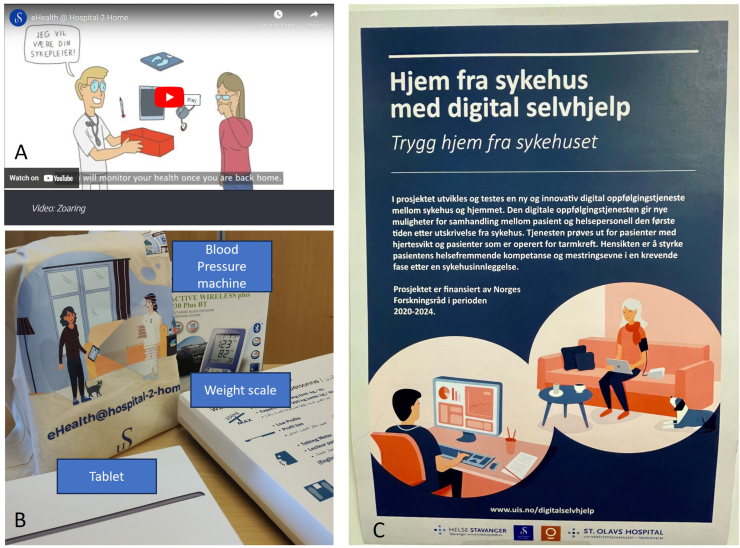
Promotional materials and equipment for participants. A) animated video explains the study: Video links: https://youtu.be/pT2NhK_jAAU (in English), https://www.youtube.com/watch?v=erkp443snwk (in Norwegian); B) Equipment and canvas bag with study logo given to participants; C) poster displayed in clinical areas (in Norwegian).

Findings demonstrated that clinical patient pathway had been considered in the design of the study. Adaptations were made, during the study, to ensure the research was specific and applicable to the local patient populations. Before recruitment started, eligibility criteria had been agreed as clinically appropriate. Patient and public involvement (provided by the UAB) was sought in the design of materials to be given to participants. The research team had considered the additional burden this study would place on participants and developed a plan to alleviate that burden. All participants were provided the required technology, device training, and received token gift cards when research equipment was returned. Researchers developed a robust marketing and research awareness plan to increase the awareness of the study with clinical teams (see people). Further research awareness was planned which included the creation of an animated film (in both English and Norwegian, a roll up poster for clinical areas, and the design and use of a study logo. Fig. 3 illustrates some promotional materials.

Despite these efforts recruitment was slow, leading the team to refine the recruitment strategy. The eligibility criteria were broadened during the recruitment of the feasibility study until the final criteria were settled on and adopted for the subsequent randomised control trial (RCT). Table 2 outlines the original and RCT eligibility criteria.

**Table 2:** EHealth@H2H eligibility criteria alterations made to improve recruitment.

	Feasibility Study		RCT	
	Inclusion	Exclusion	Inclusion	Exclusion
Age	≥18	<80	≥18	No upper limit
Illness characteristics	HF: ejection fraction ≤40 %	HF: waiting list for heart transplant, require a left assist ventricular device, life expectancy <6 months	HF: inpatient with symptomatic HF, decompensation symptoms such as: dyspnoea at rest, pulmonary congestion, and elevated NT-proBNP	HF: waiting list for heart transplant, require a left assist ventricular device, life expectancy <6 months
	CRC: receiving surgical treatment for colon or rectal cancer, CRC DUKE's class 1-3	CRC: metastatic cancer, a Clavien-Dindo surgical complication score >3, acute medical crisis	CRC surgically treated for stage I-III curable colon or rectal cancer	CRC: metastatic cancer, a Clavien-Dindo surgical complication score >3, life-threatening acute medical emergencies
Other	Speak and Write Norwegian	Mental illness, cognitive impairment, planned discharge to a nursing home, involvement in other research studies.		Severe mental illness or cognitive impairment, planned discharge to a nursing home, involvement in other research studies.

∗Eligibility Criteria as reported in feasibility protocol [21] and randomised control trial protocol [27]. HF: heart failure, CRC: colorectal cancer, RCT: randomised control trial.

Other observed project design changes made to the recruitment strategies during the feasilibity study were applied to the subsequent RCT. These included increasing the length of the intervention (based on participant feedback), regular study updates (“News”) on the study website (eHealth @ Hospital-2-Home | University of Stavanger (uis.no)), creation of social media accounts (LinkedIn and [1] @eHealthH2H (@eHealthH2H)/X) and a Norwegian news feature about the experiences of nurses and participants in the feasibility study (https://tv.nrk.no/serie/distriktsnyheter-rogaland/202302/DKRO98020923).

### 3.2 Recruitment strategies identified in the CIRRT component of *people*

The *people* component of CIRRT (Fig. 1) was considered and evaluated by answering 8 practical questions (see Table 3). Exemplar coding with data quotes is provided in supplemental material 1 (LINK to supplementary file 1).

**Table 3:** Practical questions and answers for people component of CIRRT.

Practical questions	Answers
Participants	
1 Have the participants research beliefs been considered?	User advisory board meetings to discuss the study design and intervention feasibility work to ensure how participants think and feel about the research is considered.
2 Have participant characteristics been considered?	Eligibility criteria was set to capture representative sample and participant support matched concerns of patients (e.g., intervention training).
Clinicians	
1 Is research integrated into clinical work?	Research time is funded through grants rather than routinely integrated into clinical work
2 Have the clinicians research beliefs been considered?	Clinicians' thoughts and opinions on the research project were considered and integrated.
Recruiters	
1 Are recruiters in the clinical team?	Yes, but research is not part of their normal role.
2 What is the skill level of recruiters?	Varied levels, but team included novice researchers.
Researchers	
1 What are the collaborative partnerships to be built?	Hospital managers and senior clinicians were the focus of collaborative partnerships
2 Have researchers considered research burden?	Yes, from the participant, recruiter, and clinician point of view.

**Fig. 4:**
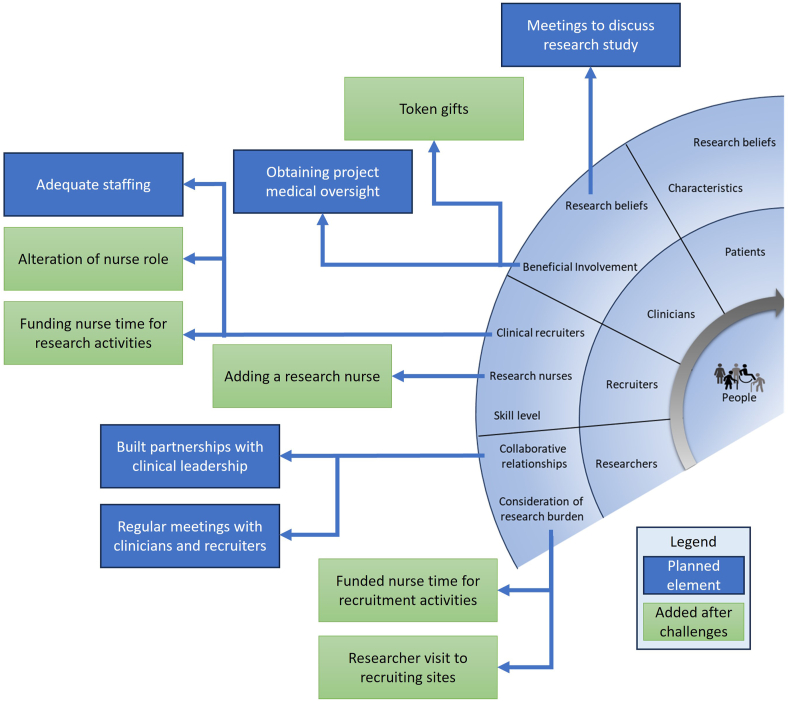
Strategies used in the CIRRT component *people* from (14). The planned strategies are in blue boxes and the added strategies are in green boxes.

Findings from the meeting minutes showed greater details around of the answers from Table 3. Coding results are highlighted in Fig. 4 to demonstrate the recruitment strategies (blue boxes) used as well as the alterations (green boxes) made due to challenges in the conduct of the study.

In the meeting minutes, the focus of their content was not on the participants research beliefs and characteristics. Instead, participant's research beliefs were reported in intervention feasibility work published elsewhere [20]. The initial recruitment strategies were reliant on clinical collaborators, who worked on the wards where possible participants were inpatients, to identify and refer eligible patients to the nurse navigators in the study. The nurse navigator role was initially defined as research consent, participant intervention training, and remote patient monitoring of participants in the intervention arm.

The eHealth@H2H project team had identified the need to build collaborative relationships with clinical teams to facilitate the identification of possible participants. Research team members instigated meetings to present the study with departmental managers, senior medical staff, and nurses in recruiting sites. Obtaining verbal agreement from clinicians and departments to collaborate by helping to identify and refer eligible participants. The team also obtained the collaboration of senior medical staff members to have clinical oversight on the project to foster collaborative partnerships.

That initial recruitment strategy was adapted due to the altered clinical staff capacities. COVID19 waves changed the workload and staff abilities to assist with patient identification and meant that nurse navigators were recalled to clinical duties. Some of the nurse navigators (those overseeing the heart failure arm) asked to be more involved in the research project and worked with the research team to alter their roles to include research screening activities. Grant funding was re-allocated to increase funded time for these nurse navigators to reflect the expansion of their role. In the colorectal nurse navigator team, the collaborative decision with the researchers was to hire a trained research nurse to aid in all recruitment activities. The researchers conducted multiple research visits to recruiting sites to fill gaps in the identification of possible research participants. Alongside developing a plan for the subsequent RCT to hire a research nurse to assume responsibility for the screening, recruitment, and training of participants in the study. These identified recruitment strategies are summarised in Fig. 4.

### 3.3 Recruitment strategies identified in the CIRRT component of *place*

The *place* component, from CIRRT (Fig. 1), was considered next and evaluated by answering 7 practical questions (see Table 4) to identity recruitment strategies and any alterations made. Exemplar coding and data quotes s in supplemental material 1 (LINK to supplementary file 1).

**Table 4:** Practical questions and answers for place component of CIRRT.

Practical questions	Answers
National/Local Oversight Institutions	
1) How is research supported?	Research positive hospitals with experience in research delivery.
2) Do healthcare and research priorities match?	Yes, Norway has stated desire to lead in digital health research.
Healthcare setting	
1) What is the clinical research culture?	Department heads and senior clinicians were all supportive of the project.
2) What are available resources?	No nationally funded research nurses.
3) Do the clinical characteristics match requirements?	Appeared to have a good match with hospital's patient population.
Community setting	
1) Was a community setting considered?	Not applicable to this study.
2) Have community partnerships been built?	Awareness of the project and the digital intervention communicated with local GP's.

**Fig. 5:**
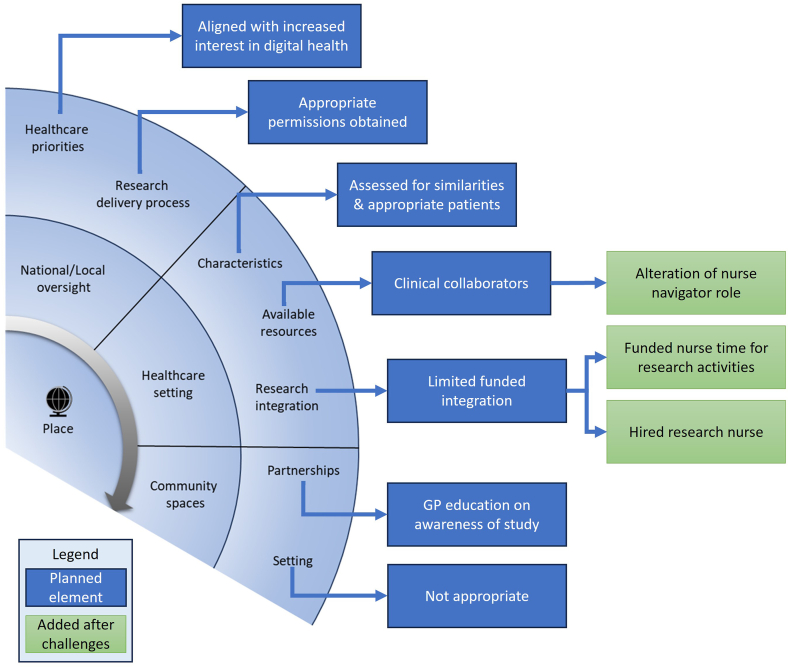
Strategies used in the recruitment component *place* from CIRRT [14]. The planned strategies are in blue boxes and the added strategies are in green boxes.

The answers in Table 4 were expanded based on the findings of the meeting minutes. The coding results are highlighted in Fig. 5 to demonstrate the recruitment strategies (blue boxes) used as well as the alterations (green boxes) made due to challenges in the conduct of the study.

It is likely that larger portions of work relating to this component occurred in earlier stages of the research project development, but some evidence was still present in meeting minutes as updates to relevant parties. The eHealthH2H project aims aligned with Norwegian healthcare priorities of exploring and expanding the use of digital technologies to improve care. Appropriate approvals were obtained meaning the project had the support of national and local oversight institutions. The recruiting centres and wards had been assessed and were thought to be the appropriate place for recruitment to occur due to existing patient care pathways and population. As outlined in *People* the department heads and clinicians were identified as clinical collaborators. While the community spaces were not thought to be the right place for recruitment to occur primary care clinicians were made aware of the project through targeted meetings and clinical awareness raised around the intervention to improve integration and acceptance of the intervention across the healthcare pathways.

During the study resources (and ability to recruit) were affected by hired nurse navigators leaving nursing roles. To offset these challenges changes to the nurse navigator role (expanding to include recruitment activities) and the hiring of additional staff (as outlined in people) was initiated, as discussed in people.

### 3.4 Impact of recruitment strategies

**Fig. 6:**
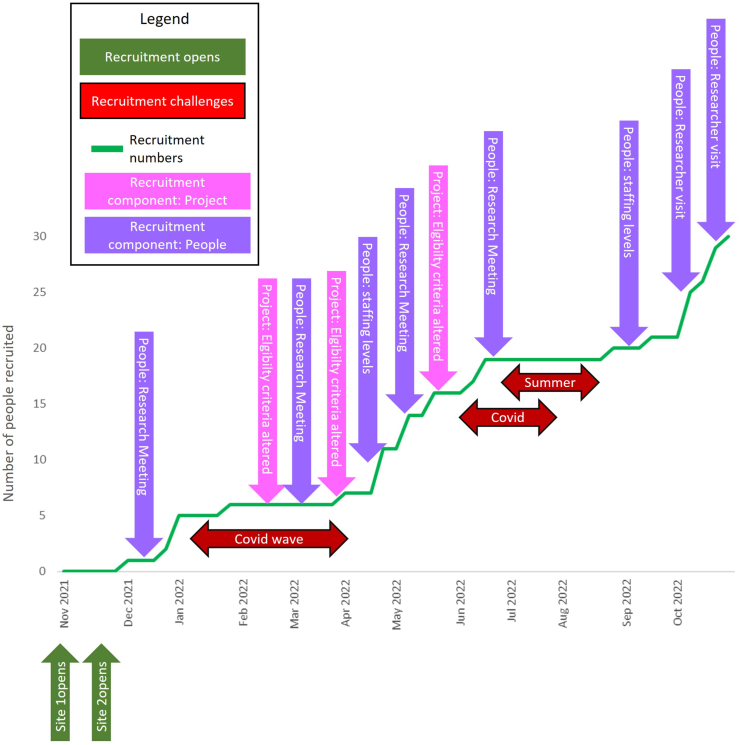
Graph showing the recruitment numbers integrated with coding of alterations to recruitment strategies in the eHealth@H2H feasibility study.

Fig. 6 shows the time matched recruitment strategies, alongside the recruitment challenges and actual recruitment numbers. While not causal, supportive actions taken by the researchers to flexibly respond to the recruitment challenges appeared to impact on recruitment success. Impact of recruitment strategies were observed within factors and elements from the people and project components of CIRRT.

Following researcher visits to recruiting sites, where they worked alongside the recruiters on screening and recruitment, there were increases in recruitment numbers. Similarly staffing levels of nurses assigned to perform the recruitment coincided with increased recruitment numbers. The impact of research meetings and changes in eligibility are less clear to observe as those changes did not always align with periods of active recruitment.

## 4 Discussion

Using CIRRT practical questions and taxonomy enabled a systematic evaluation of the complex recruitment strategies designed and adapted in the eHealth@H2H feasibility study. Recruitment strategies were identified across all components of CIRRT from meeting minutes. Key strategies around the roles and responsibilities of nurses involved in the study together with supportive research visits appear to have a positive impact on recruitment in chronic illness research.

The CIRRT was created for the purpose of guiding researchers in the design and reporting of recruitment activities [14]. This paper presents the first test of the taxonomy as a guide to report research recruitment activities. The structure of the CIRRT provided a systemic way of evaluating and then reporting changes to recruitment strategies made during the feasibility study and the subsequent RCT.

There were some occasions in the components of Project, People and Place where careful consideration was needed to ensure repetition did not occur. The nuance between these elements is important as in the component of *people* it is about capturing how the individual belief's, characteristics, roles, skills, rewards, and work practices were built into any recruitment strategy. Where in the component of *project*, the focus was on how recruitment strategies were incorporated into the study design, participant research journey and promotional material. The *place* component is focused on the how the organisational structures, characteristics, and processes in the environment where the research is to occur is considered within recruitment strategies. The authors are considering creating a more detailed guide to accompany the CIRRT.

Despite having a multi-faceted recruitment strategy, the eHealth@H2H study experienced challenges in recruitment. The largest delay to recruitment were Covid19 waves with subsequent implications on staffing levels. Collaborative partnerships and open communication with recruiting sites meant that researchers were able to respond reflexively to those recruitment challenges. Similar challenges were observed worldwide research was paused, staff redeployed, and decreased levels of clinical were reported impacts on research recruitment [[28], [29], [30], [31]].

Initial eligibility criteria (often the centre of recruitment challenges) were thought to be inclusive in the planning stage, but slow recruitment meant lead to collaborative decisions with clinicians to broaden the criteria making the research more inclusive. Narrow eligibility criteria are a frequently reported factor that impact on recruitment and generalizability of research [32,33]. The true test of the final eligibility criteria occur in the subsequent RCT [27].

Staffing and supportive researcher alterations during the feasibility study appeared to have an impact on recruitment. The eHealth@H2H research team altered the funding plan for the study to embed protected research time for nurses supporting the research to expand their roles or in the hiring of a research nurse. These changes were facilitated through grant funding, but in Norway research nurses are not government funded. The lack government funded research nurses and resultant impact on recruitment is not unique to this project with others reporting on the importance of available and funded research nurse time [34,35]. The impact of nationally supported and funded of research nurses and the national priorities embedding of protected research time for clinicians supporting research, is likely to be a key contributor to research recruitment [2].

### 4.1 Strengths and limitations

This article reports on the use of CIRRT as a tool where we retrospectively reported and evaluated research recruitment strategies. As it was used on a feasibility research study the limited sample size limits the ability toto clarify any causal relationships between research recruitment strategies to recruitment success in chronic illness research. However, CIRRT use provided a framework for the systematic reporting of recruitment strategies and proposed changes to those strategies. It also highlights the possible use of meeting minutes together with recruitment data to examine the impact of recruitment strategies. Further use and testing of this taxonomy are needed to confirm its structure with independent researchers. This may include the use of other data types, such as qualitative interviews or focus group data.

## 5 Conclusions

Recruitment challenges are common in research but are often poorly reported. The examination of research team meeting minutes from the eHealth@H2H feasibility study the enabled systematic reporting the recruitment strategies observed using the CIRRT. Recruitment strategies which appeared impactful included increasing nurses’ roles to embed research recruitment, provide adequate research staffing levels, open and regular communication with clinical teams, and research visits. Successful recruitment to chronic illness research goes beyond the alteration of eligibility criteria as highlighted using the CIRRT.

## CRediT authorship contribution statement

**Rosalynn C. Austin:** Writing – review & editing, Writing – original draft, Visualization, Formal analysis, Conceptualization. **Anne Marie Lunde Husebø:** Writing – review & editing, Project administration, Funding acquisition, Formal analysis. **Hege Wathne:** Writing – review & editing, Project administration, Investigation. **Marianne Storm:** Writing – review & editing, Project administration, Investigation, Funding acquisition, Formal analysis. **Kristin H. Urstad:** Writing – review & editing, Investigation, Funding acquisition, Formal analysis. **Ingvild Morken:** Writing – review & editing, Investigation, Funding acquisition, Formal analysis. **Bjørg Karlsen:** Writing – review & editing, Project administration, Methodology, Formal analysis.

## Funding source(s)

This work is supported by the Norwegian Research Council (Grant No. 301472) and has undergone independent peer review. The work has also received funding from the University of Stavanger, Norway for a PhD scholarship.

## Declaration of competing interest

The authors declare the following financial interests/personal relationships which may be considered as potential competing interests: Rosalynn Austin is a member of the Long-term conditions theme at the National Institute of Health and Care Research (NIHR) Applied Research Collaboration Wessex. The views expressed are those of the authors and not necessarily the National Health Service, NIHR or Department of Health and Social Care.

## Data Availability

Data will be made available on request.

## References

[1] Walters S.J., Bonacho Dos Anjos Henriques-Cadby I., Bortolami O., Flight L., Hind D., Jacques R.M. (2017). Recruitment and retention of participants in randomised controlled trials: a review of trials funded and published by the United Kingdom Health Technology Assessment Programme. BMJ Open.

[2] Kenealy T.W., Hao'uli S., Arroll B. (2015). A qualitative study of recruiting for investigations in primary care: plan, pay, minimise intermediaries and keep it simple. SAGE Open Med.

[3] McDonald A.M., Knight R.C., Campbell M.K., Entwistle V.A., Grant A.M., Cook J.A. (2006). What influences recruitment to randomised controlled trials? A review of trials funded by two UK funding agencies. Trials.

[4] Kearney A., Harman N.L., Rosala-Hallas A., Beecher C., Blazeby J.M., Bower P. (2018). Development of an online resource for recruitment research in clinical trials to organise and map current literature. Clin. Trials.

[5] Natale P., Gutman T., Howell M., Dansie K., Hawley C.M., Cho Y. (2020). Recruitment and retention in clinical trials in chronic kidney disease: report from national workshops with patients, caregivers and health professionals. Nephrol. Dial. Transplant..

[6] Donovan J.L., Rooshenas L., Jepson M., Elliott D., Wade J., Avery K. (2016). Optimising recruitment and informed consent in randomised controlled trials: the development and implementation of the Quintet Recruitment Intervention (QRI). Trials.

[7] Kasenda B., von Elm E., You J., Blümle A., Tomonaga Y., Saccilotto R. (2014). Prevalence, characteristics, and publication of discontinued randomized trials. JAMA.

[8] Miller W.R., Bakas T., Buelow J.M., Habermann B. (2013). Research involving participants with chronic diseases: overcoming recruitment obstacles. Clin. Nurse Spec..

[9] Milani S.A., Swain M., Otufowora A., Cottler L.B., Striley C.W. (2021). Willingness to participate in health research among community-dwelling middle-aged and older adults: does race/ethnicity matter?. J Racial Ethn Health Disparities.

[10] Rabinowitz Y.G., Gallagher-Thompson D. (2010). Recruitment and retention of ethnic minority elders into clinical research. Alzheimer Dis. Assoc. Disord..

[11] Elfeky A., Treweek S., Hannes K., Bruhn H., Fraser C., Gillies K. (2022). Using qualitative methods in pilot and feasibility trials to inform recruitment and retention processes in full-scale randomised trials: a qualitative evidence synthesis. BMJ Open.

[12] Donovan J.L., Jepson M., Rooshenas L., Paramasivan S., Mills N., Elliott D. (2022). Development of a new adapted QuinteT Recruitment Intervention (QRI-Two) for rapid application to RCTs underway with enrolment shortfalls-to identify previously hidden barriers and improve recruitment. Trials.

[13] Rooshenas L., Scott L.J., Blazeby J.M., Rogers C.A., Tilling K.M., Husbands S. (2019). The QuinteT Recruitment Intervention supported five randomized trials to recruit to target: a mixed-methods evaluation. J. Clin. Epidemiol..

[14] Austin R.C., Karlsen B., Richardson A., Elwyn G., Storm M., Husebø A.M.L., Urstad K.H. (2024). Taxonomy of recruitment in chronic illness research: a restricted systematic review. submitted BMC Health Services Research.

[15] Bleijenberg N., de Man-van Ginkel J.M., Trappenburg J.C.A., Ettema R.G.A., Sino C.G., Heim N. (2018). Increasing value and reducing waste by optimizing the development of complex interventions: enriching the development phase of the Medical Research Council (MRC) Framework. Int. J. Nurs. Stud..

[16] Nurse Assisted eHealth Service From Hospital to Home: Ameliorating Burden of Treatment among Patients With Non-Communicable Diseases [Internet]. Norweigan Research Council. Available from: https://prosjektbanken.forskningsradet.no/project/FORISS/301472?Kilde=FORISS&distribution=Ar&chart=bar&calcType=funding&Sprak=no&sortBy=score&sortOrder=desc&resultCount=30&offset=0&Fritekst=+Nurse+assisted+eHealth+service+from+hospital+to+home%3A+Ameliorating+burden+of+treatment+among+patients+with+non-communicable+diseases.+.

[17] Skivington K., Matthews L., Simpson S.A., Craig P., Baird J., Blazeby J.M. (2021). A new framework for developing and evaluating complex interventions: update of Medical Research Council guidance. Bmj.

[18] Morken I.M., Storm M., Søreide J.A., Urstad K.H., Karlsen B., Husebø A.M.L. (2022). Posthospitalization follow-up of patients with heart failure using eHealth solutions: restricted systematic review. J. Med. Internet Res..

[19] Husebø A.L.M., Søreide J.A., Kørner H., Storm M., Wathne H.B., Richardson A. (2024). eHealth interventions to support colorectal cancer patients' self-management after discharge from surgery—an integrative literature review. Support. Care Cancer.

[20] Wathne H., Morken I.M., Storm M., Husebø A.M.L. (2023). Designing a future eHealth service for posthospitalization self-management support in long-term illness: qualitative interview study. JMIR Hum Factors.

[21] Morken I.M., Wathne H.B., Karlsen B., Storm M., Nordfonn O.K., Gjeilo K.H. (2023). Assessing a nurse-assisted eHealth intervention posthospital discharge in adult patients with non-communicable diseases: a protocol for a feasibility study. BMJ Open.

[22] Wathne H., May C., Morken I.M., Storm M., Husebø A.M.L. (2024). Acceptability and usability of a nurse-assisted remote patient monitoring intervention for the post-hospital follow-up of patients with long-term illness: a qualitative study. International Journal of Nursing Studies Advances.

[23] World Medical Association (1996). Declaration of Helsinki (1964). BMJ.

[24] Palinkas L.A., Mendon S.J., Hamilton A.B. (2019). Innovations in mixed methods evaluations. Annu. Rev. Publ. Health.

[25] Microsoft Corporation. Microsoft Word for Microsoft 365 MSO. 2402 Build 16.0.17328.20346 ed. Redmond, WA2024.

[26] Timmermans S., Tavory I. (2012). Theory construction in qualitative research:from grounded theory to abductive analysis. Socio. Theor..

[27] Storm M., Morken I.M., Austin R.C., Nordfonn O., Wathne H.B., Urstad K.H. (2024). Evaluation of the nurse-assisted eHealth intervention ‘eHealth@Hospital-2-Home’ on self-care by patients with heart failure and colorectal cancer post-hospital discharge: protocol for a randomised controlled trial. BMC Health Serv. Res..

[28] Mirza M., Siebert S., Pratt A., Insch E., McIntosh F., Paton J. (2022). Impact of the COVID-19 pandemic on recruitment to clinical research studies in rheumatology. Muscoskel. Care.

[29] Mitchell E.J., Ahmed K., Breeman S., Cotton S., Constable L., Ferry G. (2020). It is unprecedented: trial management during the COVID-19 pandemic and beyond. Trials.

[30] Walker R.J., Jackson J.L., Asch S.M., Egede L.E. (2021). Mitigating the impact of COVID-19 on funded clinical research: crucial next steps. J. Gen. Intern. Med..

[31] Villarosa A.R., Ramjan L.M., Maneze D., George A. (2021). Conducting population health research during the COVID-19 pandemic: impacts and recommendations. Sustainability.

[32] He J., Morales D.R., Guthrie B. (2020). Exclusion rates in randomized controlled trials of treatments for physical conditions: a systematic review. Trials.

[33] Kim E.S., Uldrick T.S., Schenkel C., Bruinooge S.S., Harvey R.D., Magnuson A. (2021). Continuing to broaden eligibility criteria to make clinical trials more representative and inclusive: ASCO-friends of cancer research joint research statement. Clin. Cancer Res..

[34] McDermott C., Vennik J., Philpott C., le Conte S., Thomas M., Eyles C. (2021). Maximising recruitment to a randomised controlled trial for chronic rhinosinusitis using qualitative research methods: the MACRO conversation study. Trials.

[35] Price D., Edwards M., Carson-Stevens A., Cooper A., Davies F., Evans B. (2020). Challenges of recruiting emergency department patients to a qualitative study: a thematic analysis of researchers' experiences. BMC Med. Res. Methodol..

