# Supplementary material for: Use of the chronic illness research recruitment taxonomy to evaluate recruitment strategies in an eHealth feasibility study
Source: Contemp Clin Trials Commun. 2024 Dec 27;43:101420. doi: 10.1016/j.conctc.2024.101420 (PMC11753974; doi:10.1016/j.conctc.2024.101420)
Supplement: Multimedia component 1 [file mmc1.docx]

| CIRRT components, factors, and elements | | Exemplar Quote |
| --- | --- | --- |
| People | | |
| Participants (Patients) | Research Beliefs | - [Research team member] gave a summary from an interview with a heart failure patient who has completed the follow-up. The informant [participant] felt that he or she was involved in something important in terms of research. (MM_03/02/2022). |
|  | Characteristics | *not covered in meeting minutes, will be examined in other feasibility study publications |
| Clinicians | Research Beliefs | *Positive towards research project*   - [Research team member] reported enthusiasm for the project among both the doctors and the follow-up nurses at the Cardiology Department [site 1]. (MM_18/11/2021) - newly recruited follow-up nurses for heart failure patients at [site 1] […] positive about the project and the new role. (MM_25/03/2021) |
|  | Beneficial Involvement | *Medical oversight*   - [Research team member] has been in contact with [cardiologist] at [site 1] about relevant doctors who may be medically responsible for heart failure patients. (MM_15/04/2021). - [Research team member] have had a meeting with three senior consultants at [bowel cancer department] at [stie 1]. Two of these, [clinician names], have agreed to be medically co-responsible for the colorectal cancer patient group in the project. (MM_22/03/2021)   *Token gifts*   - [Research team member] will contact assistant heads of departments [site 1] and ask them to screen relevant patients from the patient lists. This will be a good help in patient recruitment for the appointed follow-up nurses for heart failure patients. They are offered gift cards of kr. 1000.- for this work. (MM03/06/2021)   *Added value*   - The experience of follow-up nurses has made them more independent in relation to the use of technology (MM_03/11/2022) - emphasis on the nurses feeling welcome as key partners in the team and that this can be perceived as motivating for the nurses. (MM_08/04/2021) |
| Recruiters | Clinical recruiters | *Clinical collaboration*   - [Research team member] contacts the medical officers for the bowel cancer patients to inform them of late recruitment and ask them to be observant of relevant patients who can be included. (MM_03/02/2022) - The department head and division manager have agreed to screen heart failure patients on [site 1], respectively. (MM_18/11/2021)   *Funding nurse time for research activities*   - It will be important to ensure steady recruitment to the RCT study. Brief discussion about employing the follow-up nurses in a 10 or 20% position. Then they can set aside a fixed time for project work. (MM_22/09/2022) |
|  | Research nurses | *Adding a research nurse*   - There is a desire to recruit a nurse who has the main responsibility for recruitment. (MM_20/10/2022) - [Research team member] has been in contact with Stavanger Health Research for the use of research nurses for screening patient lists and inclusion of patients. They are very busy but were due to deal with our request in a meeting on Tuesday 22.3. (MM_17-03-2022) - [Research team member] has been in contact via email with [clinical collaborator], head of section at the Department of Gastrointestinal Surgery, about the possibility of "buying" help with recruitment and, possibly, making use of a research nurse in the department. (MM_23/02/2022) |
|  | Skill level | *Research and intervention training.*   - [Research team member] has also prepared a checklist for follow-up nurses and a course description from the patient being recruited. The lists contain tasks for the research team and follow-up nurses on the patient's return journey and along the way, as well as procedures at the final project phase (collecting equipment, handing out questionnaires). (MM_11/03/2021) - The nurses raised the following points in connection with their training: Want a day where they could practice with each other in connection with recruitment – they then had to buy a day off – which is welcomed by the project team (MM_11/11/2021) - The training will take place next week, i.e. week 43; Plan for recruitment [training] and how to provide good training to patients (MM_21/10/2021) |
| Researchers | Collaborative relationships | *Build partnerships with clinicians*   - Proposal to inform the departments and doctors at the cardiology and bowel cancer departments on an ongoing basis as a reminder of the project and the recruitment of patients. (MM_03/03/2022) - [Research team member] sends an email to head of department at Cardiologist, SUS and asks the person in question to inform the cardiological doctors to be aware of which patients with heart failure can be included. (MM_03/02/2022)   *Regular meetings with clinicians and recruiters*   - [Research team member] will stop by [site 1] during the week/next week to follow up with the nurses [Gastro wards] there in connection with recruitment. (MM08/09/2022) - [Research team member] attends [site 1] every Monday to check patient lists and motivate the nurses in the cardiology department. (MM_24/03/2022) - [Research team members] have had several meetings with the follow-up nurses about their experiences from recruitment. (MM_13/01/2022) - Will gather the nurses from [site 2] who will attend a cardiology conference in Stavanger in October to discuss how to speed up recruitment. (MM_08/06/2022) |
|  | Consideration of research burden | *Alignment with clinical pathways*   - [Research team member] is in the process of investigating the best way to inform the various departments where patient recruitment will take place. (MM_02/09/2021) - [Research team members] plan a meeting with the follow-up nurses at [hospital unit] to discuss recruitment and their experience so far. […] They want to discuss the following: Experience so far, whether the nurses need help in recruitment, Distribution of work between the two nurses. (MM_27/01/2022) - There was a brief discussion about how it may be problematic for the follow-up nurses for heart failure patients who work in the intensive care unit at SUS to follow up the recruitment of patients.  In the intensive care unit, it can also be difficult to find the right patients with heart failure. (MM_27/05/2021)   *Researcher engagement*   - [Research team member] **p**ointed out the importance of us as a research group showing extra motivation and commitment in the recruitment work – "that little extra". (MM_24/03/2022) |
| Place | | |
| National/Local Oversight | Healthcare priorities | *Aligned with increased national interest in digital health*   - The project was the course of the future of health care. They [National health leaders] wanted to be informed about the development and wanted to contribute to the success of the project. (MM_27/05/2021) - [Research team member] reported from a digital meeting arranged by the Norwegian Directorate of Health, where the goal was, among other things, to improve the quality of digital follow-up of chronically ill patients. […] Proposal to invite the head of the project to a morning meeting to tell in more detail about the project. (MM_02/09/2021) |
|  | Research delivery process | *Appropriate permissions and regular updates*   - Notify the clinic directors of the initiation of recruitment and intervention. [Research team member] sends a message to the Department of Gastrological Surgery, while [Research team member] takes responsibility for providing information to the cardiology department. (MM_21/02/2021) - [Research team members] plan to apply to [governing body] for an extension of the project due COVID pandemic, understaffing due sick leave, conditions at the hospital that have delayed recruitment. (MM20/02/2022) - The follow-up nurses at [site 1] must have researcher access in order to access the patient records after discharge. This must be applied for every year. The follow-up nurses [site 2] must have the right to actualise, i.e. they have the right to access the records up to 30 days after discharge. (MM_06/05/2021) |
| Healthcare setting | Characteristics | *Assessed for appropriate patient population*   - The project was approved at St. Olav yesterday – thus it is ready for the start of the project there. (MM_02/12/2021) - Uncertain whether "slow" recruitment is due to more COVID patients hospitalized. (MM_13/01/2022) |
|  | Available resources | *Staff roles*   - [Research team member] directs an inquiry to dept. managers at [hospital unit] to hear if [nurse names] can access their patient lists. (MM29/04/2021) - 2 follow-up nurses who have left and high sickness absence at SUS which has negative consequences for recruitment. (MM_17/02/2022) - It turns out that it is difficult to use research nurses for screening patient lists and inclusion of patients as these do not have access to DIPS and patient lists. (MM_24/03/2022) - The two new 6H follow-up nurses will be [nurse name] and [nurse name]. They will receive digital training from [software staff] from Dignio on Wednesday 23.3. 15-17. (MM_17/03/2022).   *Available technology*   - There may be delays in connection with recruitment as the project currently lacks 10 i-pads in Stavanger and 5 in Trondheim due to delivery issues. (MM_11/11/2021) |
|  | Research integration | *Not observed directly in meeting minutes, but noted lack of protected time for research may hint that research is not integrated in clinical settings where recruitment occurred. Resulted in team adding a research nurse and increasing funded nurse time for research activities for subsequent RCT |
| Community Spaces | Partnerships | It is important to inform actors in the primary health service about the start of the project's trials, such as GPs, home care nurses, etc. (MM27/05/2021) |
|  | Settings | Not applicable |
| Project | | |
| Research design | Eligibility criteria | *Broad criteria*   - Believed that it would be quick to recruit relevant heart failure patients in accordance with the inclusion and exclusion criteria (MM_21/10/2021) - Proposal to have broader inclusion criteria for colorectal cancer patients and thus have a more general post-operative follow-up. (MM_24/03/2022) - [Research team member] has been in contact with [site 1] regarding slow recruitment of heart failure patients and how the speed can be increased. There is a question as to whether the inclusion criteria are too strict. (MM_10/02/2022) |
|  | Protocol | *Matched patient pathway*   - Patients who are referred to the failure outpatient clinic in Trondheim will be admitted to this clinic after approximately 3 weeks, while those referred to the outpatient clinic at SUS will not be admitted until 2 – 3 months after discharge. Most likely, this will not affect our study, as the patients will follow ordinary treatment alongside our follow-up service. (MM_14/10/2021)   *Providing technology*   - Please note that we have enough tablets and measuring equipment available to send patients home. If we do not have enough of this, we risk having to wait to recruit patients pending availability of equipment. (MM_25/02/2021)   *Project alterations*   - [Research team members] are planning a meeting with [oversight body] to inquire about the possibility of extending the project beyond this year due to delayed recruitment of patients in the main project due to the Covid pandemic, sick leave and high sickness absence at SUS. (MM_03/03/2022) |
|  | Patient and public involvement | *Regular meetings and editing patient communications*   - The brochures will now be sent to users [members of UAB] for their assessment and input. (MM_25/03/2021) |
| Participant journey | Communications | *Patient communication*   - Recommend spreading information a little throughout the hospital stay, and not collecting everything for discharge, as it quickly becomes very much information at once. (MM_25/02/2021 - Information material for patients has now been prepared and is under printing. (MM_23/09/2021) - [Research team member] has "language tested" the brochures following [SAC team member] proposal to use computer software program “Lix”. The brochures had good legibility. (MM_25/03/2021)   *Online presence*   - Website for the project has been established. (MM_31/05/2021) |
|  | Research burden | *Token gift card*   - Patients may be reimbursed for taxi expenses in connection with such arrangements for the submission of equipment. (MM_02/12/2021)   *Provide intervention technology and training*   - Please note that we have enough tablets and measuring equipment available to send patients home. If we do not have enough of this, we risk having to wait to recruit patients pending availability of equipment. (MM_25/02/2021)   *Unavoidable illness burden*   - The previously mentioned patient (see minutes from 23 February) who has not followed up the measurements at home, told [research team member] when he handed in the equipment that he was motivated at the hospital to participate in the project, but became depressed after returning home and thus failed to follow up – nor did he take prescribed any medication. (MM_03/03/2022) |
| Research Promotion | Marketing | *Animated film*   - The [animated] film was shown at the meeting and met with enthusiasm. (MM_12/08/2021)   *Logo*   - Several [research team] believed that project illustration No. 2 of those sent out yesterday is the most illustrative.  It was also pointed out that the nurse should be dressed in white and that the service user should be a woman. (MM_27/05/2021)   *Roll-up poster*   - Two roll-ups [posters] will be made for dissemination of the project: one for use in [site 2] and one for use in [site1] (MM_28/01/2021)   *News Feature*   - [Research team member] referred to [University communication team] who wants to make a news story about the project in NRK or in a newspaper through interview/demonstration of the use of Dignio with, for example, one or more follow-up nurses/project members/patients who have participated in the project. (MM_03/11/2022) |
|  | Awareness | *Regular meeting with clinicians*   - [Research team member] contacts the medical officers at the bowel cancer department and asks for the opportunity to distribute a brochure about the project while informing the patients about impending surgery. (MM_09/06/2022) - [Research team member] has received a response from [collaborating clinician] who, among other things, suggests making laminated cards that doctors can wear as a reminder in terms of recruitment. (MM_03/03/2022) |
